# A qualitative analysis of perceived barriers and motivators to physical activity in children from ethnic minority groups and white British children

**DOI:** 10.1371/journal.pone.0324781

**Published:** 2025-05-27

**Authors:** Joyce Ene Omenyo Omojor-Oche, Gavin Daniel Tempest, Florentina Hettinga, Nicola McCullogh

**Affiliations:** 1 Department of Sport Exercise and Rehabilitation, Faculty of Health and Life Sciences, Northumbria University, Newcastle upon Tyne, United Kingdom; 2 Department of Human Movement Sciences, Faculty of Behavioural and Human Movement Sciences, Vrije Universiteit, Amsterdam, Netherlands; University of Montenegro, MONTENEGRO

## Abstract

Physical inactivity is a problem worldwide despite the well-known health benefits of physical activity participation. Children from ethnic minority groups report some of the lowest levels of physical activity. In trying to address this, the voices and perspectives of children, especially children from ethnic minority groups, are often overlooked. The primary purpose of this study was to identify and compare the perceived barriers and motivators to physical activity of children from ethnic minority groups and white British children in a UK city. A semi-structured interview on physical activity participation was conducted in English with 20 children aged 8–11 years old (9 from a range of different ethnic minority groups and 11 white British children). The data were coded and analysed via inductive thematic analysis using Nvivo-12. A total of three barriers and eight motivators were identified for both groups. The barriers to physical activity participation were structure of sports; inadequate resources; and children’s social circle. The motivators were self-autonomy; self-confidence; enjoyment of physical activity; positive mental health and wellbeing; social circle; structure of sports; institutional motivators; and the introduction of more gender-balanced organised sports. Differences between groups were however noted amongst the subthemes, particularly in relation to barriers. For children from ethnic minority groups, barrier subthemes comprised parental barriers and obligations to siblings; lack of gender-balanced team sports; lack of variety of sports and lack of sporting equipment; and negative feedback from teammates. For white British children, barrier subthemes were lack of adequate play time in school and lack of financial resources. Such barriers may underpin differences in physical activity participation and should be considered in intervention design.

## 1. Introduction

Sedentary lifestyles and obesity among children are major public health problems globally, despite the immense health benefits physical activity offers [1]. In the UK, one in six deaths is related to physical inactivity and is estimated to cost the NHS about £0.9 billion and the whole of the UK about £7.4 billion annually [2]. Regulatory guidelines recommend children and adolescents between the ages of 5 and 17 years old accumulate at least an average of 60 minutes per day of moderate-to-vigorous, mostly aerobic, physical activity (PA) [3]. However, children and young adults between the ages of 10 and 24 years constitute 24% of the world’s population, of which 80% of children are not adequately active, spending over two hours daily on sedentary related activities [4]. In England, only 44.6% of children and young people between school years 1–11 (ages 5–16) achieved the Chief Medical Officers’ PA guideline of 60 minutes per day from 2020 to 2021 [5]. Similarly, almost a quarter of children aged 4–5 years starting primary school are already overweight [6]. These figures are higher for ethnic minority groups [7].

According to Love et al. [8], some of the huge disparity and inequality that exists in the amount of vigorous activity achieved by children is accrued along socioeconomic and ethnic lines. In contrast, others attribute insufficient PA or a sedentary lifestyle to rapid economic development, such as improved means of transportation [9], as well as proliferation and ease of access to technological gadgets increasing screen time viewings on laptops, televisions, phones, and other electronic devices, resulting in higher levels of physical inactivity and lower energy expenditure [10]. Furthermore, factors suggested as barriers to PA uptake in children from ethnic minority groups are parental behaviours, as most parents of ethnic minority origin have lower educational and income status and so might not know the importance of PA or have the time to take the children to PA-related activities [11,12]. This is further compounded by the fact that some parents overestimate the level of PA achieved by children [13].

Physical inactivity is a risk factor for global diseases such as colon cancer, type II diabetes, breast cancer, and cardiovascular diseases [14]. Adults and children from ethnic minority groups, when compared to the general population, have a higher incidence of physical inactivity-related diseases such as type II diabetes, cardiovascular diseases and colon cancer [15]. Available evidence suggests South Asians are 1.5 times more at risk of stroke and 9.0% vs 3.9% at risk of diabetes compared to white British individuals [16], while Afro-Caribbeans are 2.5 times more at risk of stroke compared to the total population [17]. The issue of physical inactivity amongst ethnic minority groups has been prevalent for years and is thus a public health concern [18] and neglecting to address the challenge it poses would place a higher burden on government resources as it is estimated that the NHS spent £6.1 billion on obesity-related illness in 2014/15 alone [19]. To address this public health concern, there is a need to focus PA interventions on children at an earlier age to develop and encourage life-long PA behaviours [20]. Evidence from a study conducted in Australia [21] revealed that the decline in sports and PA participation begins as early as the age of eight. Similarly, the age of decline in PA is the same for children in the UK [22]. Therefore, interventions should target young children, before the steep decline in PA and sports is observed, so that adequate PA levels are maintained [23].

Information on barriers and motivators to participation in PA in children has mostly come from parents and teachers. However, there is a need to incorporate the voices, experiences, and perceptions of children themselves, especially children from ethnic minority groups, in research and health promotion. It is important to show respectful regard for and recognition of children as active participants who have interest and capacity to discuss and impact decisions that pertain to them [24]. Controversy in the past has disputed the need for children’s voices in studies; however, the search for children’s perspectives through their inputs is a search for reality and authenticity [25]. This is one of the only means by which we can genuinely learn about a person’s (children in this instance) lived experience and fundamental nature, plus it reflects their truth, hence the value of the involvement of children in studies [26]. However, children are largely excluded from studies and interventions that pertain to them [27].

Applying the Ottawa Charter’s five-action health promotion framework has been shown to be effective as it conveys the core values of equity, participation, and empowerment [28]. Crucially, the charter suggests that programmes and interventions should be customised to the needs of participants who will be taking delivery of them, so they should include detailed planning that involves stakeholders in a needs assessment as well as implementation and evaluation [29]. Unfortunately, as we have seen, health promotion design and studies targeted at increasing children’s PA levels are largely devoid of children’s voices [30]. Also, PA research studies of children between the ages of 8 and 14 years in school settings in high-income countries are few and poorly implemented [4], while children and adolescents from ethnic minority groups receive relatively little attention [31]. Studies are further compounded by the fact that most parents overestimate the level of PA of their children [13].

Therefore, the purpose of this qualitative study was to (i) identify the perceived barriers and motivators to PA of children from ethnic minority groups and white British children in Newcastle upon Tyne, and (ii) explore any similarities or differences in the perceived barriers and motivators to PA among the children, using semi-structured interviewing and inductive thematic analysis.

## 2. Materials and methods

### 2.1. Participants

Participants were recruited from December 2022 to March 2023 through a purposive sampling approach from schools with diverse cultural and socioeconomic qualities in Newcastle upon Tyne, UK. A total of 25 schools were approached with a letter containing the study information. Schools that indicated a diverse representation of children from different ethnic minority groups were selected. Two schools consented to participate. Subsequently, the study was advertised to children between the ages of 8 and 11 years old through classroom presentations conducted by the first author and the physical education (PE) teacher. Participants were included if they were fluent in English. A total of 50 children indicated interest and they were given an information sheet and consent form to be signed by their parents/guardians.

Twenty children returned signed consent forms and participated in the study. In this study, participants were grouped into two groups based on self-reported ethnicity: ethnic minority groups and white British children, in line with the UK government recommended terminology. The demographics and self-reported ethnicities of the participants are shown in Table 1.

**Table 1:** Description of participants.

Participant (PT) ID No.	Age (years)	Gender	Ethnicity	Religion
01	9	Female	White British	Christian
02	10	Female	White British	No religion
03	10	Female	Jewish	Jewish
04	11	Female	Asian (Bangladesh)	Muslim
05	10	Female	White British	Christian
06	10	Female	Polish	Christian
07	11	Male	Asian (Saudi Arabian)	Muslim
08	10	Male	White British	No religion
09	11	Female	Asian (Filipino)	Christian
10	8	Female	White British	No religion
11	9	Male	White British	No religion
12	9	Female	White British	Christian
13	9	Female	White British	Christian
14	11	Female	White British	No religion
15	8	Female	White British	No religion
16	8	Male	White British	Christian
17	8	Male	Asian (Chinese)	Christian
18	8	Female	Asian (Arab)	Muslim
19	8	Male	Polish	Christian
20	11	Male	African (Nigerian)	Muslim

### 2.2. Ethical approval

Ethical approval (52677) was granted by the Department of Sport, Exercise and Rehabilitation Research Ethics Committee of Northumbria University.

### 2.3. Procedure

Semi-structured interviews were conducted within the space of a single week in each of the schools. Sessions took place during the children’s break time and in the school library, with the PE teacher always present. Semi-structured questions were developed and approved by the ethics committee in line with themes and study gaps derived from a previous systematic literature review [32], as well as the aim and objectives of the current study. The questions sought participants’ knowledge of PA, what they did in their leisure time, what PA they participated in at home and at school and with whom, and why. The questions comprised general topics to specifics such as motivators and barriers to PA participation, e.g., ‘Do you participate in any physical activity/games at home? If no, what are the barriers?’. All of the questions were designed to build a rapport to explore the children’s thoughts, feelings and beliefs about PA [33]. Nudging prompts such as asking them for their personal opinions or suggestions on PA were also used to allow for the flow of conversation.

This study was underpinned by the constructivist paradigm. That means the authors understood that people construct knowledge through their own interactions with the world. Thus, the perceptions of children were interpreted as their personal experiences, while at the same time, the researchers considered the ages of the children, PE teacher present during the interview, and social context (e.g., influence of their friends) in which these experiences occurred [34]. The interviews were conducted by the first author (a person of colour supported by a white British PE teacher) in English and all interviews were recorded, saved, and transcribed verbatim. The semi-structured interviews lasted between 45 and 60 minutes for each child. Each interview was audio recorded on a Sony ICD model PX240 recording device.

### 2.4. Analysis

All 20 semi-structured interviews were recorded and transcribed verbatim using an online software transcription tool (Otter.ai) and anonymised by the lead author (JO). Braun and Clarke’s [35] six phase inductive analytical approach to coding and theme development was utilised for this study [36]. Inductive thematic analysis ensures rigour in terms of identifying meaning as closely as possible from the participants themselves, as the coding process is bottom-up and data-led [37].

The lead author immersed and familiarised herself with the data by listening to the audio recording and checking against the transcription, identifying patterns and initiating the initial coding process. The 20 transcribed scripts were then exported to the NVivo software app (version 12). The coding process was conducted independently by the lead author (JO) and a second researcher (KS). The aim of this was not however to establish inter-coder reliability, especially given the inductive nature of the analysis; instead, JO and KS met afterwards for a review of potential themes and subthemes. KS acted as a “critical friend”, questioning JO’s assumptions, coding process and generated themes [38]. This was to promote reflection and bolster the further development of relevant themes [39], consistent with the standpoint that in qualitative research the researcher’s subjectivity is not a threat to accuracy but rather a resource [40] due to their active role in shaping the themes.

Next, JO and another author (NM) met for a further review of potential subthemes and themes. The two authors discussed participants’ responses, familiar patterns and what themes they represented, and then agreed on the definition and naming of themes and subthemes. This was immediately followed by a process in which JO compared the transcripts to the renamed themes and subthemes. This was to ensure rigour in the inductive thematic development process [41].

## 3. Results

### 3.1. Themes

A total of three barriers and a total of eight motivators were inductively identified for both children from ethnic minority groups and white British children (Table 2).

**Table 2:** Themes: Barriers and motivators.

	Themes	Example(s)
**Barriers**	Structure of sports	Lack of organised inclusive team sports Lack of variety of sports
	Inadequate resources	Lack of playing area Financial resources Lack of adequate play time
	Social circle	Negative feedback from teammates House chores Parental barriers
**Motivators**	Self-autonomy	Self-motivated
	Self-confidence	Builds self-confidence
	Enjoyment of PA	PA is fun Enjoyment and happiness
	Positive mental health and wellbeing	Positive mental health and wellbeing
	Social circle	Parent(s), sibling(s). friend(s) and/or family
	Structure of sports	Introduction of organised team sports Introduction of a variety of sports Introduction of gender balanced team sports.
	Institutional motivators	Introduction of green area in school Teacher(s)
	Gender-balanced organised sports	Introduction of gender-balanced team sports

### 3.2. Barriers

#### 3.2.1. Structure of sports.

Some of the children reported the lack of variety of PA opportunities (PT04 and PT06, ethnic minority groups; PT05, white British) as a barrier to participation in PA: ‘at school, outside we’ve got the little climbing thing… the first day, all the kids were on it, but now ‘til this day, only five kids are on each because all of them are getting bored’ (PT06, ethnic minority group). Also, the lack of organised team sports was reported specifically by children from ethnic minority groups (PT04 and PT20).

Maybe get everyone included properly? Like, there are some very sporty people in our class, and maybe get people that don’t feel comfortable? Because people don’t pass to them. People say they’re not good. Make them feel uncomfortable. And get them like into PE (PT04, ethnic minority group).

#### 3.2.2. Inadequate resources.

Inadequate resources in the form of insufficient financial resources (PT13, white British), insufficient play time at school (PT05, white British), unavailable neighbourhood playground (PT20, ethnic minority group), or the lack of sports equipment at home (PT09 and PT20, ethnic minority groups) could be limiting factors to participation in PA in children, especially children from ethnic minority groups: ‘I can’t really play football at home because, like the neighbourhood I’m in we don’t really have like, a stable place to play football’ (PT20).

And usually there’s not enough time because like, everyone has a game and like they run around and then when we go out like one will go in for lunch [break time doubles as mealtime] or like after break, then they don’t have time to like, go and run around or do exercise. Yeah. So, you will need more time for that (PT05, white British).

#### 3.2.3. Social circle.

As much as a social circle could be a motivator, it could also be a barrier to children’s participation in PA. This could be in the form of parents not letting children out to play or being impeded with housework (PT09, ethnic minority group), influential physically inactive friends or negative feedback from one’s social circle or teammates (PT04 and PT20, ethnic minority groups).

Sometimes, yeah… some people like, if you miss a goal, or like if you are the goalie [goalkeeper] and a ball enters, some people… they’ll just randomly yell at you and say like you’re terrible at the game. So, it won’t make you want to really play anymore (PT20, ethnic minority group).

### 3.3. Motivators

#### 3.3.1. Self-autonomy.

While most of the children had a good idea about sports, exercise, and physical activity and its correlated benefits, some of the children had more self-autonomy: ‘I usually only do football and dance sometimes at home… and I do football after school and inside school… I’m gonna start playing for the school team’ (PT01, white British); and some children were more self-motivated (PT01, PT05, PT10, PT12 and PT13, white British; PT06, ethnic minority group) than others as they had a range of activities lined up, such as walking and playing football outside. These differences were noticed between groups, with white British children generally being more deliberate about PA activities than children from ethnic minority groups.

Well, I like watching movies, but then also like when I’m in my room, I like especially painting… I just like run around with my brother and stuff. Sometimes when I go to my grandma’s, we just play football sometimes outside, but I don’t do a lot of activities outside of school unless we like go somewhere like the park or the beach. [Interviewer: How regularly do you do that?] Normally the holidays like when there’s like a half term, normally we play cricket sometimes, not all the time (PT04, ethnic minority group)

#### 3.3.2. Self-confidence.

Sports or PA for some of the children was an opportunity to demonstrate skills they had mastered and to exude confidence at a particular sport they were good at: ‘I love going on the beam because I get to like, show my balance and skills’ (PT05, white British). For others, PA helped improve their confidence: ‘Football? Makes me feel a lot bigger… Like, makes me feel like taller’ (PT17, ethnic minority group).

#### 3.3.3. Enjoyment of PA.

The children were more likely to participate in PA if the PA was fun (PT03, PT04 and PT06, ethnic minority groups; PT05 and PT15, white British): “Because I do have swimming lessons and we get to do handstands in the water that I really like and somersaults and stuff like that. And I love just jumping in’ (PT03, ethnic minority group); ‘it’s fun’ (PT05 and PT15, white British); ‘at school… I go on the climbing thing a lot… The climb thing just makes us want to climb; it makes us walk more… I still always love it. And I always go on it’ (PT06, ethnic minority group).

#### 3.3.4. Positive mental health and wellbeing.

As part of the nudging questions, the children were asked to discuss motivating factors for participation in PA. Some of the children spoke of the positive impact it had on their mental wellness (PT03, PT04 and PT06, ethnic minority groups; PT14, white British), while some of the children said PA helped clear their mind and helped them feel good (PT04, ethnic minority group; PT10 and PT12, white British).

Like I do dancing. It keeps me calm, even though like, because I do lots of dancing because I dance for six hours, but I do seven classes… Like it’s a really nice place to be. And it’s calming me down because it’s hard to be with three brothers because two of them are dogs, and one of them’s my birth brother (PT10, white British).

#### 3.3.5. Social circle.

Social factors play a crucial role in children participating in PA, for instance in the form of parents participating (PT01 and PT14, white British; PT03 and PT06, ethnic minority groups): ‘I like walking with my dad because we also go in jokes and stuff in the morning if we’re not busy. And me and my dad went to go like on a walk and stargaze at the same time’ (PT03, ethnic minority group). Parents played a further role in taking children for PA (PT02, PT05 and PT08, white British): ‘My mom takes me [to gymnastics] … I go with my sister’ (PT02, white British). Other social facilitators included children playing with siblings (PT04, PT07, PT17 and PT18, ethnic minority groups) or playing with friends (PT01, PT05, PT10, PT12, PT13 and PT15, white British; PT04, PT18, PT19 and PT20, ethnic minority groups): ‘[my three friends and I] always like go to dance and come back together. And… we usually just like, have fun and drive there. And we always have fun at dance’ (PT10, white British).

#### 3.3.6. Structure of sports.

How team sports are structured, especially in schools, can have a positive or negative impact on how children participate in PA, particularly children from ethnic minority groups. The children were asked to suggest factors that would encourage PA uptake. Some recommended the introduction of a variety of sports (PT02, PT08, PT10, PT11, PT14, PT15 and PT16, white British; PT06 and PT09, ethnic minority groups): ‘trampoline’ (PT08, white British); ‘I feel like we can have like a gymnastics club’ (PT10, white British); ‘probably tennis… basketball’ (PT11, white British). More organised team sports were also recommended (PT04, PT07 and PT09, ethnic minority groups), for instance:

My only suggestion would be like to make the teams fair like in sports. Like when there’s a game going on… because sometimes like, it gets a bit annoying… for the children, like when they don’t enjoy playing with the teams (PT07, ethnic minority group).

#### 3.3.7. Institutional motivators.

Schools and teachers play important roles in PA levels in children as children spend most of their waking hours in school. Some of the children noted the positive impact the PE teacher had in organising the football team and how it had made them want to participate in PA (PT20, ethnic minority group), and some suggested there were also positive impacts of structures such as natural green areas (PT06, ethnic minority group) and indoor places where they could play during bad weather (PT02, PT05 and PT14, white British).

And so, it was just recently because once Miss [PE teacher] announced the football team, like she said that you have to be a good sportsman. So, people were becoming nicer… So, I wanted to play more. And other people also wanted to play. And after I wanted to play more, and I was more like, dedicated in the game, because I didn’t really let up on the goal anymore. And so now everyone thinks I’m a good goalkeeper (PT20, ethnic minority group).

There used to be a garden club, you know, it would be so fun. I wish it was back. Those people used to do the garden club there. But the plants have just broken down. So now it’s gone now (PT06, ethnic minority group).

#### 3.3.8. Introduction of more gender-balanced organised sports.

Many of the children reported their love for football, however some of the girls suggested a need for gender-balanced team sports as playing with a team full of boys could be rough or it just affected their confidence (PT04, PT06 and PT09, ethnic minority groups): ‘I like playing I just don’t like playing with all the boys because they will tackle you and stuff’ (PT03, ethnic minority group).

There was no girls, so it was only me. So that’s why I got a bit stressed out but… when the next time we’re out there were five girls and seriously, I was like, ‘Oh my, I want to be friends with them’ because they were so kind. They were hoping I’d come. They were looking at us. Like, ‘I hope you have an amazing time here. We gonna help you’ (PT06, ethnic minority group).

### 3.4. Subthemes

Comparatively, a total of four barrier subthemes were identified for children from ethnic minority groups and a total of two barrier subthemes were identified for white British children. The barriers identified for children from ethnic minority groups that did not feature in the feedback from white British children include parental barriers and obligations to siblings; lack of gender-balanced team sports; a lack of variety of sports and sporting equipment; and negative feedback from teammates. On the other hand, barriers to PA participation identified by white British children that did not feature in feedback from ethnic minority groups were a lack of adequate play time in school and a lack of financial resources, suggesting some difference between the groups.

In contrast, six motivator subthemes were shared by children from ethnic minority groups and white British children: self-autonomy; self-confidence; friends; family; introduction of a variety of sports; and enjoyment of PA and wellbeing. Children from ethnic minority groups reported one further motivator: the introduction of gender-balanced team sports.

## 4. Discussion

Through a series of interviews with children, this study sought to investigate (i) the perceived barriers and motivators to PA participation amongst children from ethnic minority groups and white British children in Newcastle upon Tyne, and (ii) any similarities or differences in the perceived barriers and motivators to PA among the children. The findings suggest barriers and motivators occur at different levels, from the individual themselves to external influences beyond their control. This brings to the fore Urie Bronfenbrenner’s socio-ecological theory.

The socio-ecological theory contributes significantly to the theoretical understandings of PA behaviour in children [42]. It identifies the different levels or factors that could impact PA participation in children, including children from ethnic minority groups, which includes the intrapersonal level (self-autonomy, self-confidence, enjoyment of PA and wellbeing), the interpersonal level (parents, friends, family and teachers), and the institution (schools, introduction of a variety of sports). Understanding the PA behaviour of children from ethnic minority groups and/or developing a PA intervention could therefore be guided by the socio-ecological model [43].

At an intrapersonal level, the outcomes from the current study suggested a lot of the children are knowledgeable about PA and sports and the benefits they present. However, when asked about activities performed during their leisure time, white British children appeared to be more intentional in their PA behaviours, showed skills and exuded confidence in skills they had mastered over time. The white British children had goals, and some sought to improve on their competence irrespective of their perceived ability, having planned activities such as going for walks, playing football and playing on the trampoline. In contrast, some of the children from ethnic minority groups were motivated by playing with siblings while others outlined sedentary activities such as watching movies and playing in their rooms or were simply not allowed to go out to play. These findings are consistent with the discussion of Wigfield et al. [44] that there exist differences in how children and adolescents achieve motivation in pursuit of their PA-related tasks. Some children pursue tasks with great persistence whilst others avoid them. Such observations exist along gender, cultural and ethnic differences.

Researching motivation and how it can be applied to any aspect of PA in children and adolescents is crucial to understanding differences in behavioural, cognitive, and emotional participation; some children exhibit higher levels of engagement while others display boredom, lack of interest, or completely withdraw [45]. Six out of the 20 children in this study (PT01, PT05, PT10, PT12 and PT13, white British; PT06, ethnic minority group) clearly showed initiative and self-motivation in being active and did not need much external prompting. However, child PT04 from an ethnic minority group said, ‘but I don’t do a lot of activities outside of school’ despite the benefits she experiences from being active: ‘It helps me get… rid of like if I have anything on my mind.’

The children were asked to make suggestions about how to improve the PA experience and to list barriers and motivators to PA participation. Nine of them (PT02, PT08, PT10, PT11, PT14, PT15 and PT16, white British; PT06 and PT09, ethnic minority groups) suggested the introduction of a variety of sports as an important factor. This was because it translated into enjoyment of PA and wellbeing for most of them. Available evidence over time suggests there is a positive correlation between children’s level of enjoyment and participating in PE or PA [46]. The higher the level of enjoyment of a particular sport a child experiences, the higher the level of participation in PE and PA [47]. Furthermore, enjoyment of PA and PE may be key to long-term motivation, as children who enjoy PA are likely to adopt a physically active lifestyle [48]. The findings of the current study demonstrate the importance of enjoyment as a motivational factor for PA in children from ethnic minority groups. It is therefore crucial to incorporate it into future interventions, recommendations and guidelines. The possibility of tailoring opportunities to the children’s needs, interests and social environment, allowing for choice, might motivate them and increase participation in PA [49,50].

The next level of influence in the socio-ecological model is the interpersonal level. The interpersonal level concerns the relationships closest to the individual, which exert the most influence on them. These include parents, friends, teachers, siblings and other members of the family [51]. The white British children performed some of their activities with their parents which suggests a deliberate act on the part of the parents, thus suggesting parental awareness and support for PA, unlike some children from ethnic minority groups [52]. This could be as a result of differences in culture, and how parents from ethnic minority groups perceive PA, its purpose and correlated benefits [53]. Parents play a very crucial role in PA uptake in children and adolescents [54]. Furthermore, the feedback from this study highlights the fact that children, as individuals or groups, experienced differences in barriers and motivators unique to them; there is therefore a need to consider these factors in the context in which they occur, based on how they impact decisions to be physically active, if we are to effectively motivate and promote PA behavioural change in children [55]. These factors include but are not limited to social support, PA attitude, negative feelings when active as a result of previous PA experience, and extrinsic barriers such as household responsibilities and limited access to playground or sports equipment at home [56]. Finally, playing with friends, which was one of the important motivating factors, should be taken into account in the design and implementation of PA interventions, as data suggest that greater self-consciousness and peer pressure begin to develop from the age of 6 years [57]. Evidence suggests children would perform better if they had positive feedback from their social circle, however, negative feedback from their social circle will affect their esteem, thus negatively impacting their PA motivation and experience [58]. This is consistent with Harter’s competence motivation theory which suggests perception of competence is correlated to children’s participation in PA [59,60].

Returning to the socio-ecological model, the next level of influence is the institution level (school), within which we have included the role of the teacher. The school environment is crucial in the PA levels of children. This is because children and adolescents spend most of their waking time at school [61], and therefore a large proportion of PA occurs in the school environment. School PA participation also addressed most of the PA barriers experienced by children from ethnic minority groups, such as parental and household responsibilities, lack of sporting equipment and lack of a conducive sporting environment at home.

The role of the PE teacher and the school in establishing a positive PE and PA experience can significantly influence children to choose a healthy, active lifestyle [62]. Furthermore, responsibility for the introduction of gender balance in organised sports – a suggestion made by the children in this study – might also fall on teachers. While some of the children from ethnic minority groups complained of negative feedback from their teammates during PA activities, having a knowledgeable teacher or PE teacher with a grounding in motivational theories can make all the difference as one of the students (PT20, ethnic minority group) reported the positive impact the PE teacher’s motivational statement had on them and how most of them had become more interested in team sports.

Finally, at a community/policy level, the introduction of free local sports opportunities for all children to increase their PA engagement would be valuable as financial resources may also be a barrier [63].

The current study is one of the few studies of PA participation that focuses on children from diverse ethnic backgrounds in the UK. Future research addressing inclusion and low PA uptake in children from ethnic minority groups should consider these findings, for example by acknowledging the importance of enjoyment and choice in addition to introducing exercise as a generic recommendation or intervention, and by acknowledging the potential differences in social factors. In terms of the practical implications of the findings, it is critical to provide a leeway for tailoring generic PA interventions to address specific needs of children from ethnic minority groups to address the variances in barriers and motivators associated with the levels of the socio-ecological model [64]. This includes consideration at the community/policy level, which may be challenging due to the likely need for governmental funding to support the design and delivery of PA initiatives which address factors such as children’s self-motivation for PA participation as well as offering opportunities for varied and enjoyable PA. However, given the public health benefits of children’s participation in PA, including the potential long-term cost savings for the NHS, this is certainly a possibility that policymakers may wish to explore. Initial teacher training programmes and continuing professional development activities may also be extended to further upskill teachers in facilitating positive school-based PA experiences to promote children’s long-term PA participation.

### 4.1. Limitations

This study aimed to represent the diverse ethnic groups that reside in Newcastle upon Tyne, however, many of the 25 schools contacted were unable to participate due to time constraints, thereby limiting a wider participant recruitment. With a small sample of 20 children from only two schools ultimately participating in the research, and the potential differences between children whose parents returned (40%) and did not return (60%) consent forms (e.g., in demographics and prosocial behaviour scores [65]), it must be acknowledged that the participants may not be fully representative of 8- to 11-year-olds even within the geographical area of focus. Despite this difficulty, participants from a range of ethnic minority groups were recruited. Future work should aim for a broad participant base to capture diverse perspectives.

Whilst grouping the children into the broad categories of ethnic minority groups and white British children was consistent with the aim and focus of this early exploratory study, it would be valuable for future research to explore the perceptions of children from specific ethnic groups in greater depth to obtain a more detailed picture.

### 4.2. Conclusion

This study provides insight on the motivators and barriers to PA participation in children from ethnic minority groups living in Newcastle upon Tyne. The positive or negative impact of a child’s social circle on PA cannot be overemphasised, as factors such as parents’ PA behaviour and support are crucial, as can be friends participating in the same sport. On the flip side, negative feedback from teammates could also discourage children, especially children from ethnic minority groups, from participating in PA. Furthermore, one of the motivating factors that stood out for all children was the introduction of variety in the sports available to them, which suggests the large impact of this factor on PA for white British children as well as children from ethnic minority groups. Social barriers were however more prevalent in children from ethnic minority groups, and while the school (institution) played a positive role in PA participation for all children, the school environment may be especially important for children from ethnic minority groups in cases where factors such as parental and household responsibilities pose a barrier to PA participation at home. Therefore, there is a need for government and charity organisations to provide an enabling and thriving environment and support to schools to carry out these responsibilities.

Comparatively, barriers amongst the ethnic minority groups that did not feature in feedback from white British children were parental barriers and obligations to siblings; a lack of gender-balanced team sports; a lack of variety of sports and a lack of sporting equipment; and negative feedback from teammates. On the other hand, barriers to PA participation reported by white British children which did not feature in feedback from ethnic minority groups were a lack of adequate play time in school and a lack of financial resources, suggesting there are some differences between the groups which would benefit from further study, and which should be considered when designing PA interventions. Children’s perspectives are an important element of intervention design as children can identify their own barriers and motivators to participation in PA.
